# Stimulation of angiogenesis resulting from cooperation between macrophages and MDA-MB-231 breast cancer cells: proposed molecular mechanism and effect of tetrathiomolybdate

**DOI:** 10.1186/1471-2407-10-375

**Published:** 2010-07-17

**Authors:** Ulrich Joimel, Caroline Gest, Jeannette Soria, Linda-Louise Pritchard, Jérôme Alexandre, Marc Laurent, Emmanuel Blot, Lionel Cazin, Jean-Pierre Vannier, Rémi Varin, Hong Li, Claudine Soria

**Affiliations:** 1 Laboratoire M.E.R.C.I - EA 3829, Faculté de Médecine et de Pharmacie, Université de Rouen, 22 Bd Gambetta, 76183 Rouen cedex, France; 2 Service et Laboratoire d'Oncologie Médicale de L'Hôtel Dieu de Paris, Paris, France; 3 UMRS 872 INSERM, Université Pierre et Marie Curie-Paris 6 and Université Paris Descartes, Equipe 18, Centre de Recherche des Cordeliers, Paris, France; 4 Université Paris-Sud and CNRS FRE 3239, IAL, 7 rue Guy Moquet, BP8, 94801 Villejuif Cedex, France; 5 Université Paris Descartes, Hôpital Hôtel-Dieu, AP-HP Paris, France

## Abstract

**Background:**

Infiltration by macrophages (Mφ) indicates a poor prognosis in breast cancers, in particular by inducing angiogenesis. Our study aimed 1) to investigate the mechanism by which cooperation between Mφ and aggressive breast cancer cells (MDA-MB-231) induces angiogenesis; 2) to examine the effect of tetrathiomolybdate (TM) on this angiogenic activity.

**Methods:**

Mφ coincubated with MDA-MB-231 were used as a model to mimic the inflammatory microenvironment. Angiogenesis induced by the culture media was tested in the chick chorioallantoic membrane (CAM). Mφ phenotype was evaluated by 1) expression of the M1 marker CD80, and secretion of interleukin 10 (IL-10), an M2 marker; 2) capacity to secrete Tumour Necrosis Factor α (TNFα) when stimulated by lipopolysaccharide/interferon γ (LPS/IFNγ); 3) ability to induce MDA-MB-231 apoptosis. To explore the molecular mechanisms involved, cytokine profiles of conditioned media from MDA-MB-231, Mφ and the coculture were characterised by an antibody cytokine array. All experiments were carried out both in presence and in absence of TM.

**Results:**

Incubation of Mφ with MDA-MB-231 induced a pro-angiogenic effect in the CAM. It emerged that the angiogenic activity of the coculture is due to the capacity of Mφ to switch from M1 Mφ towards M2, probably due to an increase in Macrophage Colony Stimulating Factor. This M1-M2 switch was shown by a decreased expression of CD80 upon LPS/IFNγ stimulation, an increased secretion of IL-10, a decreased secretion of TNFα in response to LPS/IFNγ and an inability to potentiate apoptosis. At the molecular level, the angiogenic activity of the coculture medium can be explained by the secretion of CXC chemokines/ELR^+ ^and CC chemokines. Although TM did not modify either the M2 phenotype in the coculture or the profile of the secreted chemokines, it did decrease the angiogenic activity of the coculture medium, suggesting that TM inhibited angiogenic activity by interfering with the endothelial cell signalling induced by these chemokines.

**Conclusions:**

Cooperation between Mφ and MDA-MB-231 transformed M1 Mφ to an angiogenic, M2 phenotype, attested by secretion of CXC chemokines/ELR^+ ^and CC chemokines. TM inhibited this coculture-induced increase in angiogenic activity, without affecting either Mφ phenotype or cytokine secretion profiles.

## Background

It has become clear that analysis of tumour stroma is of crucial importance to better understanding cancer progression. Cancer cell growth and invasion of surrounding tissues, as well as the metastatic process itself, require the support of the cancer stroma.

Macrophages (Mφ) form the major component of the inflammatory infiltrate observed in tumours [1]. They exhibit a distinct phenotype and are termed Tumour-Associated Macrophages (TAM) [2]. Monocytes enter tumours through blood vessels, and a number of tumour-derived chemoattractants are thought to ensure this ongoing recruitment [3,4]. This is also supported by the observation that the levels of chemoattractant proteins in tumours correlate positively with the numbers of TAMs present in these tumours [5]. Classically, it has been accepted that fully activated Mφ have the potential to inhibit tumour development. TAMs that express an M1 phenotype are characterized by a proinflammatory cytokine profile and expression of Major Histocompatibility Complex molecules. They are capable of killing tumour cells mainly by secreting inflammatory cytokines such as Tumour Necrosis Factor α (TNFα). Although associated with better prognosis, immune responses to a tumour are often weak and not able to destroy the tumour completely [6], suggesting that tumours have developed mechanisms for escaping immune surveillance. Further, the presence of Mφ in breast cancer is associated with a poor prognosis [7-10] due to the role of TAMs as promoters of tumour progression and invasion. Increasing evidence also indicates that TAMs enhance angiogenesis, contributing to cancer cell proliferation and dissemination [11-14]. This could explain the positive relationship between high levels of TAMs and breast cancer aggressiveness and their correlation with high vessel density as reported in the literature [15,16].

We have previously shown that the deleterious role of Mφ in cancer progression is due to the cooperation of monocytes with cancer cells [17]. The modifications of Mφ functions in tumours could be explained by a switch of M1 Mφ to M2 Mφ, which have protumoral properties, including promotion of angiogenesis, matrix remodelling and suppression of adaptive immunity [18,19]. This indicates a functional plasticity and an in situ Mφ reprogramming. The biological mechanism for this switch remains controversial. For Hagemann *et al. *[20,21] and Greten *et al. *[22], the malignant epithelial cancer cells drive nuclear factor kappa-light-chain-enhancer of activated B cells (NF-κB) activation by TAMs in a way that maintains their immunosuppressive M2 phenotype. Consequently, the blockage of NF-κB might be expected to switch the M2 Mφ to an M1 phenotype. Hence these studies would predict a potential anti-tumour effect of NF-κB inhibitors by the restoration of M1 immunity, providing a cytotoxic activity. However, the role of NF-κB in this switch is contested by others. Saccani *et al. *[23] and Bohuslav J *et al. *[24] conversely proposed that during the M1 to M2 Mφ switch, an inactivation of NF-κB occurs, explaining the absence of TNFα production when Mφ are stimulated by lipopolysaccharide (LPS). This could be due to a massive nuclear localization of the p50 NF-κB protein resulting in p50/p50 homodimers. Since the p50 homodimers lack the transactivation domain, they compete with the canonical p65/p50 heterodimers for the NF-κB binding sites on the inflammatory gene promoters, thereby blocking p65/p50 promoter binding and gene transcription.

Thus, although the relationship between inflammation and cancer aggressivity is widely accepted, many of the molecular and cellular mechanisms mediating this relationship remain unclear. The current study was designed to determine the role of the cooperation between Mφ and the aggressive breast cancer cells MDA-MB-231 in angiogenesis promotion. To elucidate the mechanism, we mimicked the inflammatory tumour environment by a coincubation of MDA-MB-231 cells with Mφ. Angiogenic activity of the coculture medium was compared with the culture medium from both MDA-MB-231 and Mφ cultured individually in the chick chorioallantoic membrane (CAM) model.

We first determined that the cooperation between Mφ and cancer cells led to a switch of M1 Mφ to the M2 phenotype, known to be angiogenic. Then we explored the potential molecular mechanisms responsible for this angiogenic activity. Results indicate that a wide repertoire of chemokines (CXC-ELR^+ ^and CC-chemokines) may drive the neoangiogenesis induced by the cooperative effects of cancer cells and Mφ. Finally, tetrathiomolybdate (TM), an oral copper-depleting agent that has been shown to inhibit tumour-cell-induced angiogenesis [25-27] was tested in our model of cooperation between Mφ and MDA-MB-231. This was important to check because the effect of TM has not previously been analysed in this context of inflammation in cancers.

## Methods

### Cell Culture and Reagents

MDA-MB-231 cells were maintained in RPMI 1640 medium (Eurobio) supplemented with 100 U/ml penicillin (Eurobio), 100 μg/ml streptomycin (Eurobio), 2 mM L-glutamine (Eurobio) and 10% heat-inactivated foetal calf serum (FCS, Eurobio). All incubations were carried out in a humidified atmosphere at 37°C and 5% CO_2_. LPS was purchased from Sigma-Aldrich and human recombinant interferon γ (IFNγ) from R&D Systems. TM is a gift from Professor F. Sécheresse, Institut Lavoisier (CNRS UMR 8637, Versailles).

### Preparation of Mφ

Human monocytes were isolated as described [28]. Briefly, monocytes were isolated from buffy coats using lymphocyte separation medium (Eurobio). Peripheral blood mononuclear cells were washed three times with phosphate-buffered saline (PBS, Eurobio) and allowed to adhere to Primaria™ cell culture flasks (Becton Dickinson) for 1 h at 37°C. Nonadherent cells were removed. Monocytes were then incubated in AIM-V^® ^medium (Gibco) for 7 days, as recommended for long-term cultivation of human Mφ [29]. Medium was changed, and nonadherent cells discarded, every 2 days. We found that all monocyte-derived cells isolated this way express the marker CD14, which can be used to distinguish them from cancer cells.

### Coculture of MDA-MB-231 cancer cells with Mφ

Primary human monocyte-derived Mφ were detached using a 30 min incubation with Accutase™ enzyme cell detachment medium (eBioscience). 25 × 10^3 ^MDA-MB-231 cells were plated in 96-well culture dishes (7.8 × 10^4 ^cells/cm^2^) and allowed to adhere for 6 h at 37°C. More than 95% of the cells adhered to the wells. Then the same number of Mφ was added, and cocultures were maintained for 5 days in RPMI 1640 medium supplemented with 100 U/ml penicillin, 100 μg/ml streptomycin, 2 mM L-glutamine and 10% heat-inactivated FCS. Spent medium was replaced by fresh medium every 24 h. Control groups consisted of 2.5 × 10^4 ^MDA-MB-231 alone and of 2.5 × 10^4 ^Mφ alone cultured under the same conditions. Where indicated, after 5 days of incubation the cultures were treated with 1 μg/ml LPS and 5 ng/ml IFNγ for 24 h to activate the Mφ. Cultures approached but did not reach confluence during this time.

### *In vivo *angiogenesis assays using the CAM model

All MDA-MB-231 and Mφ cultures were carried out both in presence and in absence of 5 μM TM (final concentration). The CAM assay was performed as described by others [30], using conditioned media collected from the cocultures and from individual MDA-MB-231 and Mφ cultures during the final 24 h of incubation, and stored at -80°C until use. Briefly, fertilized chick eggs (White Leghorn) purchased from the Ferme Avicole HAAS, Kaltenhouse, France, were incubated for 4 days at 37°C and a relative humidity of 80%. During this period, the eggs were positioned with the pointed end down and rotated several times. After this incubation, the shells were cracked open and the embryo eggs placed in plastic culture dishes (Merck-Eurolab) according to an established shell-less culture technique exposing the CAM to a direct access for experimental manipulation. At day 6 of embryonic development, angiogenic areas were circled with a silicon ring (Weber Métaux). To induce angiogenesis, 33 μl of either the culture medium to be tested or recombinant human basic Fibroblast Growth Factor (bFGF) (R&D Systems) (positive control) were next placed inside the rings on successive days for 3 days. Treated areas were photographed and the extent of angiogenesis evaluated 24 h after the last treatment: the total number of vessels which had sprouted from the primary vessels of the CAM and the total length of the neoangiogenesis were determined using Saisam software (Microvision Instruments).

### Characterization of Mφ by immunofluorescence analysis

Cultures and cocultures were carried out as described above, except that each cell population was seeded into 8-well Lab-Tek™ II -CC2™ chambers (Nunc) at a density of 5.7 × 10^4 ^cells/cm^2^. Five days later, all cultures were treated with 1 μg/ml LPS and 5 ng/ml IFNγ for 24 h before washing and labelling. Mφ were identified by examining the expression of CD14, which is a differentiation antigen expressed by monocytes and Mφ, and of CD80, which is a marker of M1 Mφ [31]. Expression of these molecules was evaluated by immunofluorescence both before and after activation with 1 μg/ml LPS and 5 ng/ml IFNγ for 24 h. Breast cancer cells were identified by cytokeratin 19 (CK 19) expression.

Briefly, cells were fixed for 15 min in 4% paraformaldehyde/PBS, then washed and incubated for 30 minutes with PBS containing 5% bovine serum albumin (BSA). For Mφ identification, samples were incubated with rabbit anti-CD14 polyclonal antibodies at 2 μg/ml (Abcam) or biotinylated monoclonal anti-human CD80 at 2.5 μg/ml (Ancell Corporation) for 60 min at room temperature. For identification of breast cancer cells, they were then washed in PBS/1% BSA before incubation with monoclonal anti-human CK 19 (Dako) diluted 1:50 in PBS/1% BSA. Following washing in PBS with 1% BSA, cells were incubated with, respectively, DyLight 488™-conjugated goat anti-rabbit IgG secondary antibody or DyLight 549™-conjugated streptavidin (Thermoscientific) for 60 minutes at room temperature or else Texas-Red labelled goat anti-mouse Ig diluted 1:2000 (AbD Serotec). After final washing, cover slips were mounted with mounting medium (Immunoconcepts). Samples were photographed using a Leica DM 5500B. Controls prepared without primary antibodies confirmed that, under the conditions used, background fluorescence was negligible.

### Ability of LPS + IFNγ-activated Mφ to secrete TNFα

For these experiments, cells cultured as described above were activated on day 5 with 1 μg/ml LPS and 5 ng/ml IFNγ for 24 h. These 24-h culture supernatants were then collected and tested for TNFα. Concentrations of TNFα within the supernatants were determined by human TNFα Enzyme-Linked Immunosorbent Assay (ELISA) Ready-Set-go! (eBioscience) using the standard protocol given by the manufacturer, and compared to those of supernatants from non-activated control cultures. TNFα concentrations are expressed in pg/ml. 3 independent experiments were carried out both in presence and in absence of 5 μM TM.

### Ability of Mφ to induce apoptosis in cocultures

Mφ and MDA-MB-231 cells were cultured separately and together (coculture) as indicated above for 5 days, in presence or absence of 5 μM TM. Apoptosis was measured using the ELISAPLUS cell death detection kit (Roche Diagnostics). Briefly, apoptosis-induced Desoxy-Ribonucleic Acid (DNA) fragmentation was evaluated by quantifying the histone-complexed DNA fragments (nucleosomes) found in the cytoplasm, as indicated by the manufacturer. Results are expressed as the adjusted absorbance, A405 minus A490.

### Ability of Mφ to secrete interleukin 10 (IL-10)

For these experiments, supernatants were harvested on day 5 from cells cultured as described above. These 24-h culture supernatants were then tested for IL-10. Concentrations of IL-10 within the supernatants were determined by human IL-10 ELISA Ready-Set-go! (eBioscience) using the standard protocol given by the manufacturer. IL-10 concentrations are expressed in pg/ml. 3 independent experiments were carried out both in presence and in absence of 5 μM TM.

### Cytokine array

The Human Cytokine Antibody Array III kit (RayBiotech) was used to evaluate 42 different cytokines: Epithelial Neutrophil-Activating peptide-78 (ENA-78), Granulocyte Colony-Stimulating Factor (GCSF), Granulocyte-Macrophage Colony Stimulating Factor (GM-CSF), Growth related oncoprotein (GRO), GROα, I-309, IL-1α, IL-1β, IL-2, IL-3, IL-4, IL-5, IL-6, IL-7, IL-8, IL-10, IL-12 p40p70, IL-13, IL-15, IFNγ, Monocyte Chemotactic Protein-1 (MCP-1), MCP-2, MCP-3, Macrophage Colony Stimulating Factor (MCSF), Macrophage-Derived Chemokine (MDC), Monokine induced by IFNγ (MIG), Macrophage Inflammatory Protein-1α (MIP-1α), Regulated on Activation Normal T cell Expressed and Secreted (RANTES), human Stem Cell Factor (SCF), Stromal-Derived Factor-1 (SDF-1), Thymus and Activation-Regulated Chemokine (TARC), Transforming Growth Factor-β1 (TGF-β1), TNFα, TNF-β, Epidermal Growth Factor (EGF), Insulin-like Growth Factor-I (IGF-I), Angiogenin, Oncostatin M, Thrombopoietin, Vascular Endothelial Growth Factor (VEGF), Platelet-Derived Growth Factor-BB (PDGF BB) and Leptin. GRO detects CXC Ligand 1 (CXCL1), CXCL2 and CXCL3; GROα detects only CXCL1. VEGF detects VEGF-165 and VEGF-121.

Briefly, 1 ml of undiluted supernatants harvested on day 5 from cells cultured as described above were incubated with arrayed antibody membranes, which were then exposed to the specific biotin-antibody cocktail, following the manufacturer's instructions. Signals were detected using labelled streptavidin by exposure on X-ray films. The relative amount of each cytokine present in the coculture medium is presented as the fold increase of the spot intensity in the coculture medium as compared to that of the culture medium of either Mφ cultured alone (for cytokines which were not secreted by MDA-MB-231), or MDA-MB-231 cultured alone (for cytokines which were not secreted by Mφ), or else the sum of the two (for cytokines secreted separately by both Mφ and MDA-MB-231 when cultured individually). The area density of the spots was evaluated using imageJ (written in Java) which was downloaded from the National Centre for Biotechnology Information. Signals were normalized against the positive controls across membranes. Cytokine array experiments were carried out in duplicate.

## Results

### Angiogenic activity of the Mφ + MDA-MB-231 coculture medium in the CAM

**Figure 1:**
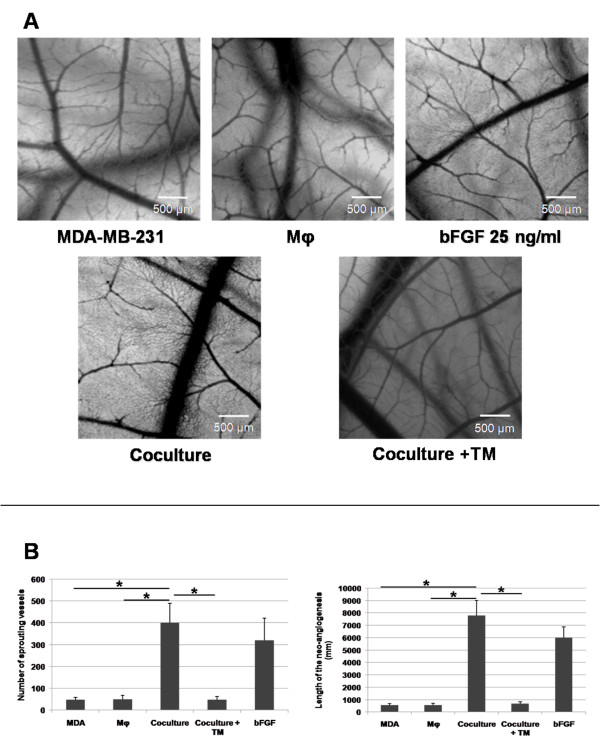
**Effect of the cooperation between Mφ and MDA-MB-231 cells on vascularisation of the CAM**. MDA-MB-231 cells were seeded at a density of 7.8 × 10^4 ^cells/cm^2^, with (coculture) or without the same number of Mφ. Spent medium was replaced by fresh medium every day. After 5 days of incubation, the conditioned media corresponding to the final 24 h of culture were tested for their angiogenic activity as follows: each day, for 3 days, 33 μL of these media were introduced into silicon rings placed on CAMs from 6-day-old chick embryos. bFGF (25 ng/ml) was used as positive control. **Panel A**: Treated areas were photographed 24 h after the last treatment. Note the increase in the CAM of a denser secondary capillary network induced by the coculture medium, in comparison to that induced by conditioned medium from either MDA-MB-231 or Mφ cultured alone. This secondary capillary network was inhibited by the addition of 5 μM TM during the coculture. **Panel B**: total number of vessels that sprouted from the primary vessels of the CAM and total length of the neoangiogenesis. Mean of 3 independent experiments ± SEM. * p < 0.05, Mann-Whitney U test.

We found that the conditioned medium from cocultures of Mφ + MDA-MB-231 induced a more potent angiogenic response in the CAM than that induced by conditioned medium from either Mφ or MDA-MB-231 cells cultured alone, as shown by the formation of a secondary dense thin capillary network only after treatment with coculture medium (Figure 1A). Indeed, both the number and the cumulative length of the capillaries that sprouted from CAM vessels were significantly increased by the coculture medium (Figure 1-B) (p < 0.05). The positive control was bFGF.

### Cell densities on day 0 and after 5 days of culture (day 5)

When plating the cells on day 0, more than 95% were adherent (both cancer cells and Mφ). We next determined the relative proportions of the two cell types in the coculture after 5 days, because MDA-MB-231 is a cell line which proliferates in culture, whereas Mφ, which are primary cells, do not. We found that in 5 days the number of MDA-MB-231 increased 5.3-fold (standard error of the mean (SEM), ± 0.35) (not shown). In contrast, Mφ numbers did not change during the 5 days of incubation, and fewer than 10% were found detached, floating in the supernatants. After the 5 days of coculture, the ratio of CK 19^+ ^(cancer cells) to Mφ was also determined. It was found that 15% of the cells were Mφ, and 85% MDA-MB-231 (not shown).

### Modifications of Mφ phenotype induced by coculture

**Figure 2:**
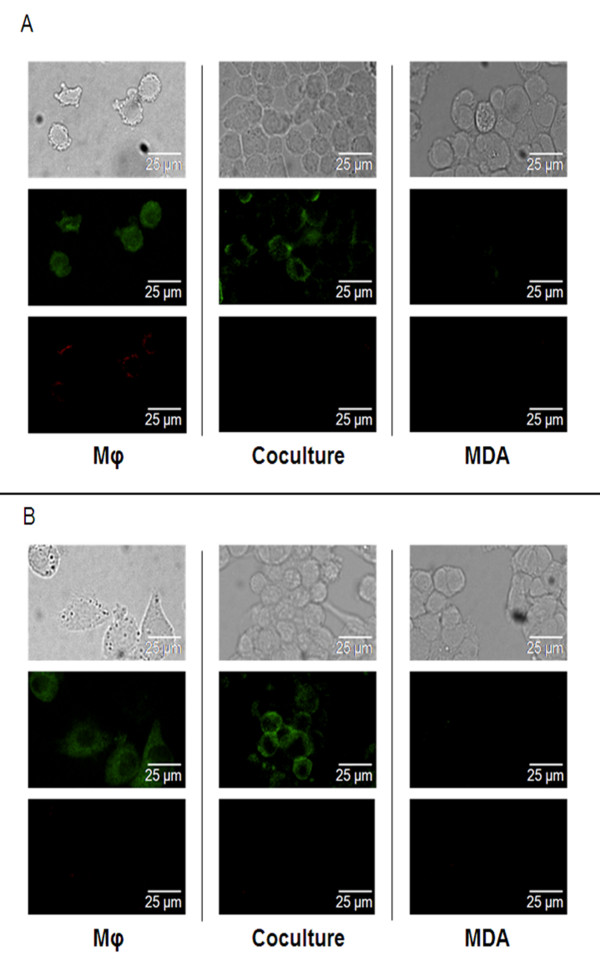
**Immunolabelling of Mφ by the monocyte/Mφ marker CD14 and the M1 Mφ marker CD80**. Mφ were seeded at a density of 5.7 × 10^4 ^cells/cm ^2^, with (coculture) or without the same number of MDA-MB-231 cells. In each case, the culture medium was replaced daily. 5 days later, all cultures were treated with 1 μg/ml LPS and 5 ng/ml IFNγ for 24 h before washing and labelling. CD14 fluorescent labelling is green, CD80 is red. **Panel A**: without TM, it can be seen that CD80 was expressed on activated Mφ cultured alone and was not detectable on Mφ in the cocultures. **Panel B**: in presence of TM, no CD80-positive cells were found in any of the cultures.

Because the switch of M1 Mφ to the M2 phenotype is known to induce angiogenesis, we investigated the possibility of an M1-M2 switch when Mφ were coincubated with MDA-MB-231 cells. Immunolabelling of Mφ cultured in presence or absence of MDA-MB-231 showed that all cells isolated as Mφ are CD14 positive. These CD14^+ ^cells were found to express CD80 only when activated by LPS/IFNγ, indicating that activation could stimulate them to differentiate towards an M1 phenotype. However, in the cocultures of Mφ with MDA-MB-231 cells, expression of CD80 by these activated Mφ was lost (Figure 2).

### Effects of coculture on apoptotic activity, TNFα and IL-10 secretion

**Figure 3:**
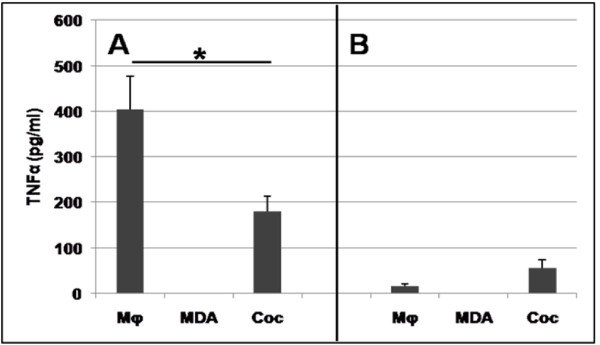
**Secretion of TNFα into the culture medium of LPS and IFNγ-treated Mφ, MDA-MB-231 and cocultures**. Cultures were incubated in the absence (**A**) or presence (**B**) of 5 μM TM, the corresponding medium being renewed daily. In all cases, cells were stimulated on day 5 with 1 μg/ml LPS and 5 ng/ml IFNγ for 24 h. Supernatants from these stimulated Mφ, MDA-MB-231 and cocultures were then tested for the presence of TNFα by ELISA. Mean ± SEM of 3 independent experiments. * p < 0.05 between Mφ and coculture without TM; p < 0.01 between Mφ without TM and Mφ with TM; p < 0.05 between coculture without and with TM. Mann-Whitney test. TNFα was not detected in the supernatants of non-activated cells, as also observed by cytokine array.

Without stimulation, Mφ did not secrete detectable amounts of TNFα. In contrast, as shown in Figure 3, panel A, Mφ activated by LPS/IFNγ secreted a large amount of TNFα. However, when these Mφ were cocultured with MDA-MB-231, a decreased secretion of TNFα was observed (180 pg/ml in the coculture medium versus 400 pg/ml in the Mφ culture). This reduction of TNFα secretion by activated Mφ is indicative of a change in Mφ phenotype from M1 to M2.

**Figure 4:**
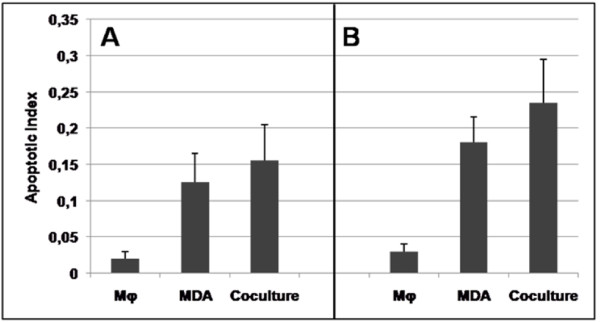
**Apoptosis of Mφ, MDA-MB-231 breast cancer cells, and coculture cells**. Cells were incubated for 5 days in the absence (**A**) or presence (**B**) of TM (5 μM, final concentration), the corresponding medium being renewed daily. Cell Death Detection ELISA PLUS enzyme immunoassay (Roche Applied Science) was used for the quantitative *in vitro *determination of cytoplasmic histone-associated DNA fragments (mono- and oligonucleosomes) in the cytoplasmic fractions of cells from these 5-day cultures. The results are expressed as the absorbance at 405 nm minus the absorbance at 492 nm. The apoptosis index measured in the coculture was equivalent to the sum of the index measured in cultures of MDA-MB-231 alone plus that of Mφ alone. Mean ± SEM of 3 independent experiments.

We also found that the coculture of Mφ with cancer cells did not potentiate the apoptotic activity of the Mφ. Indeed, as shown in Figure 4, panel A, the apoptosis index measured in the coculture was equivalent to the sum of the index measured in cultures of MDA-MB-231 alone plus that of Mφ alone.

**Figure 5:**
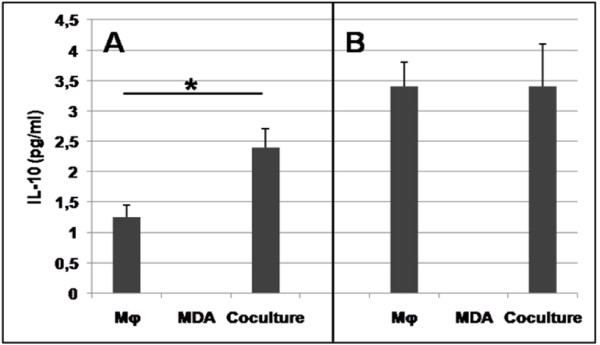
**IL-10 secretion by Mφ cultured in the absence or presence of MDA-MB-231**. Cells were incubated for 5 days in the absence (**A**) or presence (**B**) of TM (5 μM, final concentration), the corresponding medium being renewed daily; then culture media corresponding to the final 24 h of incubation were tested for IL-10 by ELISA. No IL-10 was detected in medium from MDA-MB-231 cultured alone. Mean ± SEM of 3 independent experiments. * p < 0.05 between Mφ without TM and coculture without TM; p < 0.05 between Mφ without TM and Mφ with TM.

All these results suggest that Mφ incubated with MDA-MB 231 lose their M1 phenotype. Furthermore, a switch toward the M2 phenotype is suggested by the cooperation of Mφ with MDA-MB-231, because the secretion of IL-10 increased approximately twofold in the coculture medium, compared to medium from Mφ cultured alone (Figure 5).

### Molecular mechanism underlying the angiogenic activity induced by the coculture medium

**Figure 6:**
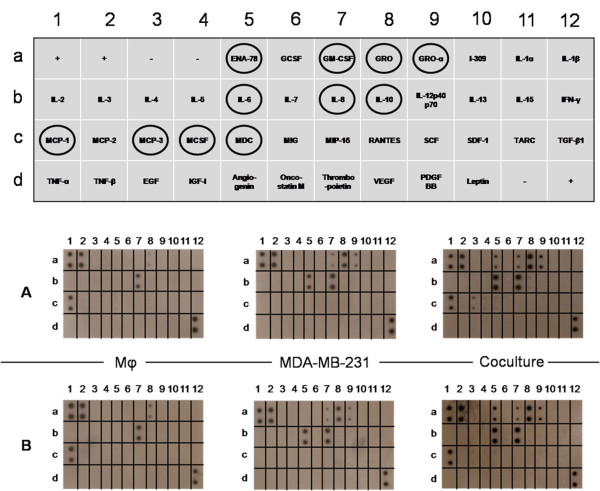
**Cytokines secreted by Mφ, by MDA-MB-231 cells and by the coculture**. The assay was performed after 5 days of culture in the absence (**A**) or presence (**B**) of 5 μM TM (final concentration), the corresponding medium being renewed daily. Culture supernatants corresponding to the final 24 h of incubation were then collected and assayed for cytokine production using the Human Cytokine Antibody Array III kit (RayBiotech). Ellipses indicate the coordinates of the secreted cytokines and chemokines. Films were developed from array membranes following incubation with supernatants from either Mφ cultures, MDA-MB-231 cells or the coculture. Positive controls are located at positions A1, A2, D12. Negative controls are located at positions A3, A4 and D11. This experiment was repeated once with similar results.

To explore the molecular mechanism responsible for the angiogenic activity that arises from the cooperation between Mφ and cancer cells, we used an antibody-cytokine array to analyse the secretion of cytokines and chemokines. Results of this analysis (shown in Figure 6 and Table 1) revealed an increase in the coculture medium, in comparison to the conditioned medium of both Mφ alone and MDA-MB-231 alone, of the following mediators: CXC chemokines with an ELR motif, chemokines that are known to be angiogenic (such as GRO, GROα, ENA-78 and IL-8) and chemoattractant chemokines, such as MCP-1 and MCP-3, that can lead to a positive feed-back. An increased secretion of M-CSF was also found and could be responsible for the switch from M1 to M2. Fold changes of cytokine levels in the coculture medium as compared to culture medium of Mφ, MDA-MB-231 or the sum of Mφ + MDA-MB-231 cultured separately are presented in Table 2.

**Table 1:** Modifications of chemokines induced by coculturing Mφ with MDA-MB-231 cells

Chemokine	Expressed in		Overexpressed in coculture	Family	Function
				
	Mφ	MDA			
**ENA 78 (CXCL5)**	**-**	**-**	**+**	CXC chemokine with ELR motif	- Pro-angiogenic activity - Neutrophil chemotaxis - Monocyte migration

**GM-CSF (CSF2)**	**-**	**+**	**+**	Growth factor	- Stimulates stem cells to produce granulocytes and monocytes - Enhances monocytic migration via RhoA and integrin activation, and via MMP expression

**GRO (CXCL1, 2, 3)**	**+**	**+**	**+**	CXC chemokine with ELR motif	- Angiogenic activity - Increased migration of PBMC/monocytes

**GROα (CXCL1)**	**-**	**+**	**+**	CXC chemokine with ELR motif	- Angiogenic activity - Increased migration of PBMC/monocytes

**IL-6**	**-**	**+**	**+**	Inflammatory cytokine	- Inflammatory cytokine with a well-documented role in cancer - Recruitment of myelo-monocytes

**IL-8 (CXCL8)**	**+**	**+**	**+**	CXC chemokine with ELR motif	- Migration of neutrophils - Important role in tumour growth, angiogenesis, and metastasis

**IL-10**	**-**	**-**	**+**	- Anti-inflammatory cytokine	- Anti-inflammatory cytokine produced by M2 Mφ

**MCP-1 (CCL2)**	**+**	**-**	**+**	CC chemokine	- Recruitment of monocytes to sites of injury

**MCP-3 (CCL7)**	**-**	**-**	**+**	CC chemokine	- Inflammatory cytokine

**MCSF (CSF1)**	**-**	**-**	**+**	Growth factor	- Facilitates monocyte survival, monocyte-to- Mφ conversion, Mφ proliferation, M1 to M2 switch

**MDC (CCL22)**	**-**	**-**	**+**	CC chemokine	-Chemotactic for monocytes

**Table 2:** Fold increase in cytokine secretion by coculture of monocytes and MDA-MB-231, as compared to the secretion by the two types of cells cultured separately

Cytokines	Fold change
**Group 1: cytokines secreted by Mφ and not by MDA-MB-231:** **Ratio coculture medium/Mφ medium**	

MCP-1	**1.45**

**Group 2: cytokines secreted by MDA-MB-231 and not by Mφ** **Ratio: coculture medium/MDA-MB-231 medium**	

GM-CSF	**2.82**

Groα	**2.96**

IL-6	**2.68**

**Group 3: cytokines secreted by both Mφ and MDA-MB-231** **Ratio coculture/(Mφ medium cultured separately + MDA-MB-231 medium cultured separately)**	

Gro	**1.80**

IL-8	**2.35**

**Group 4: cytokines secreted only in the coculture medium**	

ENA-78	

IL-10	

MCP-3	

In every case, the difference of spot intensity between duplicate determinations was <10%. The mean of the 4 spots (2 membranes × 2 spots per membrane) was used for determining the intensity of expression of each cytokine.

A faint spot indicating a low level of IL-10 production was also observed in the coculture medium, which corroborates the ELISA results suggesting that Mφ in the coculture acquired the M2 phenotype.

### Effect of TM on the angiogenic activity induced by the coculture

As shown in Figure 1, panels A and B, TM inhibited the angiogenesis that was induced in the CAM by the coculture medium: both the total number of sprouting vessels and the cumulative length of those vessels induced by TM-containing coculture medium were similar to those measured with conditioned medium from either monocytes or MDA-MB-231 cells cultured separately in the absence of TM. The observed difference in the effects of coculturing the cells in presence versus absence of TM is highly significant.

We next analysed the mechanism by which TM inhibited the angiogenic activity of the coculture medium. Because the M2 Mφ phenotype was found to be causally implicated in the angiogenic activity of the coculture medium, we first hypothesized that TM should be able to inhibit the M1-M2 switch observed during the coculture. However, TM did not elicit a change from M2 to M1 phenotype, as shown by the following results: 1) in the coculture medium, whether in the absence or in the presence of TM, CD14+ Mφ activated by LPS/IFNγ failed to express CD80; and what's more, the expression of CD80 on Mφ cultured alone was prevented by the addition of TM to the culture medium, as shown in Figure 2A and 2B. 2) TM inhibited by 80% the secretion of TNFα in LPS/IFNγ-stimulated Mφ cultured either alone or with MDA-MB-231, suggestive of an M2 phenotype (Figure 3). Likewise, TM did not potentiate the apoptotic activity in the coculture, where the apoptosis level was equivalent to the sum of the levels seen in the individual cultures: MDA-MB-231 alone plus Mφ alone (Figure 4A and 4B). These results indicate that TM does not act by "reversing" the Mφ phenotype (M2 to M1), consistent with our observation that IL-10 secretion was increased in TM-treated Mφ (Figure 5).

Finally, we also tested the action of TM on the secretion of cytokines and chemokines that is induced by the cooperation between cancer cells and Mφ. No changes were observed when TM was added during the coculture (Figure 6, comparison between panel A without TM and panel B in the presence of TM). The ratio of cytokines secreted in the presence of TM in comparison to its absence did not vary, as the fold change was between 0.85 and 1.15.

## Discussion

Mφ constitute the most abundant immune cell population present in the tumour microenvironment [1]. In many cancers, including breast cancers, it is now widely accepted that the presence of Mφ is associated with a poor prognosis. An increase in angiogenesis has been considered to be one of the major causes of the deleterious effect of Mφ in tumours. For example, the density of blood microvessels correlates with the extent of Mφ infiltration in breast cancer [11]; hence we hypothesized that there was a cooperation between Mφ and cancer cells to induce this angiogenic process. We therefore developed a model which mimics this cooperation by culturing Mφ *in vitro *with the aggressive breast cancer cells MDA-MB-231, analysed the consequences of this cooperation, and investigated the molecular mechanism mediating this angiogenic pathway. The Mφ population we tested was derived from blood monocytes and thus, at least theoretically, might be anticipated to contain some dendritic cells as well. Although we do not formally exclude this possibility, our protocol for Mφ preparation does not favour differentiation into dendritic cells. Indeed, neither the classical stimuli for dendritic cell differentiation (IL4 plus GM-CSF [32,33]) nor more recently reported contributory agents (IFNα, TNFα, IL-15, thymic stromal lymphopoietin, Toll-like receptor ligands [34]) were present during the 7-day culture period used to prepare Mφ for testing. Removal of nonadherent cells during Mφ generation further disfavours dendritic cell differentiation, given that immature dendritic cells have been shown to be nonadherent cells [35]. Moreover, MDA-MB-231 cells secrete IL-6 (Table 1), which is known to switch the differentiation of monocytes from dendritic cells to Mφ [36].

We found that the coculture medium induced greater angiogenic activity than did the culture medium of either Mφ or MDA-MB-231 alone in the CAM model; this enhanced angiogenic activity was evidenced by the formation of a secondary capillary network in the CAM with increases in both total number of capillaries and total length of sprouted vessels. The increased angiogenesis was still seen when coculture medium was diluted 1:2 in order to normalize for the number of cells participating in the secretion of angiogenic factors (not shown).

Next, we confirmed that the coculture of Mφ with MDA-MB-231 cells induced a switch of M1 Mφ to an M2 phenotype as shown 1) by the great reduction of CD80 expression under stimulating conditions using LPS and IFNγ on CD14 positive cells (Mφ) - the fact that LPS markedly potentiated the expression of CD80 by Mφ has also been observed by Foss *et al. *[37], 2) by the reduction of TNFα secretion by LPS + IFNγ activated Mφ, suggestive of a reduction in the number of M1 Mφ during the coculture, and 3) by the increased secretion of IL-10 into the coculture medium, pointing to a switch toward an M2 phenotype. Interestingly, this switch occurred despite the fact that, as shown by the antibody cytokine array, MDA-MB-231 cultured alone produce IL-6, which is an inflammatory cytokine (Figure 4). The M2 phenotype in our model was also characterized by its functional properties: a weak killing activity, and an increase in angiogenesis in the CAM model.

Our results are in good agreement with a number of studies showing that TAMs, which exhibit a predominantly M2-like phenotype [7], can elicit increased angiogenic activity. However, the molecular mechanism responsible for this angiogenic activity was not totally elucidated. To address the molecular mechanism of the angiogenesis pathway, we investigated the production of pro-inflammatory chemokines and cytokines in cocultures of MDA-MB-231 cells and Mφ, in comparison to the secretion by MDA-MB-231 or Mφ cultured separately. Our data (Figure 6 and Table 1) show that the coculture medium contains a number of potent angiogenic factors, as detected by a cytokine-antibodies array. Among the cytokines that are secreted in large amounts into the coculture medium in comparison with the culture medium of resting Mφ or of MDA-MB-231, we observed a great increase in CXC chemokines with the three amino acids (Glu-Leu-Arg/ELR) immediately amino-terminal to the CXC motif (ELR^+^), which are known to be pro-angiogenic [38]. In particular, an important secretion of the GRO chemokines CXCL1/GROα, CXCL2/GROβ, CXCL3/GROγ, as well as CXCL5/ENA-78 and CXCL8/IL-8 was noted. All these chemokines play a significant role in mediating angiogenic activity during tumourigenesis in a variety of cancers [39-44] and have been shown to be of great importance in tumour progression [45,46].

Interestingly, in addition to their angiogenic activity, it has been reported that these chemokines also play a role in monocyte recruitment in inflammation, since the blood monocytes express chemokine receptors, including CXCR2. Besides other functions, the interactions of these receptors with their chemokines induce monocyte arrest and transmigration through the endothelium, which is one of the earliest steps in monocyte recruitment, thereby efficiently regulating inflammation [47]. Therefore our data are suggestive of an autocrine loop, because these chemokines are secreted by Mφ in cooperation with cancer cells, and they also participate in monocyte recruitment.

In addition, ENA-78 was reported to be an attractant for neutrophils [48], which can also be involved in tumour growth and metastasis [49].

An increase in the proinflammatory cytokine IL-6 was also observed in the coculture medium, whereas a decrease in the secretion of this cytokine would have been expected due to the M2 phenotype of the Mφ in the coculture. This can be explained by the observation of a constitutive secretion of IL-6 by the MDA-MB-231 cells, which could be responsible for an autocrine loop for IL-6 secretion, as has been proposed for colon cancer [50]. In other words, the observed increase in IL-6 secretion in the cocultures may be produced by the cancer cells themselves.

Our antibody array data also indicated an increase in M-CSF concentration in the coculture medium in comparison to the conditioned media from both Mφ alone and MDA-MB-231 alone. This increased secretion could elicit neovascularisation in breast cancer [5,12] and could also contribute to the M1 to M2 switch, since M-CSF-treated monocytes express a substantial part of the M2 transcriptome [51].

We next investigated the effect of TM on the angiogenesis induced by the cooperation between Mφ and the MDA-MB-231 cancer cells. Several studies emphasize that TM reduces the angiogenic activity induced by tumours [52-58], which is why TM has been proposed in the therapy of cancers. However, the effect of TM has not previously been tested when cancer cells are incubated with Mφ, which represents a more physiological context: cancer associated with inflammation. We firstly demonstrated that the action of TM cannot be explained by a reversion of the angiogenic Mφ phenotype (M2 to M1), since CD80 was not detected on cells cultured in presence of TM. We also determined that the addition of TM led to a reduction of LPS/IFNγ-induced TNFα secretion (decrease of more than 80%) and an increase of IL-10 secretion in cocultured cells, and did not increase the apoptotic potential of Mφ. Pan *et al. *[52] have proposed that TM inhibits angiogenesis through suppression of the NF-κB signalling cascade. Given that TNFα secretion is NF-κB dependent, our TNFα results (Figure 3) are consistent with this interpretation, and seem to indicate that TM, like the coculture, induces a switch from M1 to M2. They also suggest that defective activation of NF-κB could lead to an M2 phenotype.

Finally, we show that, in our model, TM, which inhibits the angiogenic activity of the coculture medium, does not modify the secretion of cytokines and chemokines that is induced by the coculture. Therefore, we rather propose that TM blocks the cell signalling induced by the action of angiogenic chemokines on endothelial cells. This would be in good agreement with the studies of Donate *et al. *[26] and Juarez *et al. *[59], who showed that TM attenuates angiogenesis through the inhibition of superoxide dismutase.

## Conclusions

In summary, based on the results and rationale presented here, we conclude that the cooperation between Mφ and the aggressive breast cancer cells MDA-MB-231 transformed M1 Mφ to an angiogenic, M2 phenotype. This coculture-induced increase in angiogenic activity, attested by an increased secretion of CXC chemokines, was found to be inhibited by TM. However, TM did not modify the cytokine secretion profile, which suggests that the TM-mediated anti-angiogenic activity may be due to defective cell signalling by these angiogenic chemokines at the level of their endothelial cell targets.

## List of abbreviations used

bFGF: basic Fibroblast Growth Factor; CAM: chick chorioallantoic membrane; CK: cytokeratin; DNA: Deoxy-Ribonucleic Acid; EGF: Epidermal Growth Factor; ELISA: Enzyme-Linked Immunosorbent Assay; ENA-78: Epithelial Neutrophil-Activating peptide 78; FCS: foetal calf serum; GCSF: Granulocyte Colony-Stimulating Factor; GM-CSF: Granulocyte-Macrophage Colony Stimulating Factor; GRO: Growth Related Oncogene; HIF: Hypoxia-inducible Factor; IGF-I: Insulin-like Growth Factor-I; IL: interleukin; IFNγ: interferon γ; LPS: lipopolysaccharide; Mφ: macrophage; MCP-1: Monocyte Chemotactic Protein-1; MCSF: Macrophage Colony Stimulating Factor; MDC: Macrophage-Derived Chemokine; MIG: Monokine induced by IFNγ; MIP-1α: Macrophage Inflammatory Protein-1α; NF-κB: nuclear factor kappa-light-chain-enhancer of activated B cells; PBS: phosphate-buffered saline; PDGF BB: Platelet-Derived Growth Factor-BB; RANTES: Regulated on Activation Normal T cell Expressed and Secreted; SCF: human Stem Cell Factor; SDF-1: Stromal-Derived Factor-1; SEM: standard error of the mean; TAM: Tumour-Associated Macrophages; TARC: Thymus and Activation-Regulated Chemokine; TGF-β1: Transforming Growth Factor-β1; TM: tetrathiomolybdate; TNFα: Tumour Necrosis Factor α; VEGF: Vascular Endothelial Growth Factor.

## Competing interests

The authors declare that they have no competing interests.

## Authors' contributions

UJ was responsible for this study, designing and executing experiments, interpreting the results and contributing to drafting the manuscript.

CG participated in the analysis and interpretation of the results, was involved in writing the manuscript and provided assistance to UJ for the experiments.

JS participated in study development and critically reviewed the data, contributing to interpretation of the results and writing the manuscript.

LLP contributed to analysis and interpretation of the results and was extensively involved in writing the manuscript.

JA was involved in drafting the manuscript and revising it for important intellectual content.

ML provided assistance to UJ for the experiments.

EB participated in the design of this study, in analysis and interpretation of the results, and in development of the study methods.

LC, JPV and RV provided general intellectual support and participated in data discussions.

HL and CS were responsible for this study, participating in its design and development, analyzing and interpreting the results, drafting the manuscript and coordinating and overseeing all stages of revision of the manuscript.

All authors read and approved the manuscript.

## Pre-publication history

The pre-publication history for this paper can be accessed here:

http://www.biomedcentral.com/1471-2407/10/375/prepub
